# The Polygenic Risk Score for Parkinson’s Disease Is Associated with Becoming a Medical Doctor or Dentist

**DOI:** 10.3390/genes16040384

**Published:** 2025-03-28

**Authors:** Hikaru Takeuchi, Ryuta Kawashima

**Affiliations:** 1 Division of Developmental Cognitive Neuroscience, Institute of Development, Aging and Cancer, Tohoku University, Sendai 980-8575, Japan; 2 Department of Advanced Brain Science, Institute of Development, Aging and Cancer, Tohoku University, Sendai 980-8575, Japan; ryuta@tohoku.ac.jp

**Keywords:** polygenic risk score, Parkinson’s disease, medical doctor

## Abstract

Background/Objectives: Multiple independent studies indicate an association between the occupations of medical doctors and dentists and the risk of Parkinson’s disease (PD). This study tried to evaluate the associations between a polygenic risk score (PRS) for Parkinson’s disease (PD) and medical career (medical doctor/dentist). Methods: This population-based cross-sectional study used data from the UK Biobank. A total of 92,566 and 166,531 men and women aged 38–73 years, recruited between 2006 and 2010, were included in the analyses of job history and current job, respectively (separate samples). Odds risks for the jobs of medical doctors and dentists were estimated using logistic regression. A PRS of polymorphisms previously shown to predict PD best was constructed and associated with the job history of medical doctors/dentists in the first analysis and with current medical doctor/dentist jobs in the second analysis after regressing out confounding variables. Results: A high PD PRS was associated with employment as a medical doctor or dentist for both the 92,566 individuals with job history data, with an increase of 1 standardized deviation (*p* = 0.006), and current employment as medical doctors/dentists among the 166,531 individuals without job history data but with current job data. Furthermore, a higher PD PRS was associated with higher education in both samples. Conclusions: These results suggest that PD has shared genetic routes with a propensity for higher education and becoming medical doctors/dentists.

## 1. Introduction

Parkinson’s disease (PD) is a progressive neurodegenerative disorder caused primarily by the degeneration of dopaminergic neurons in the substantia nigra [1]. The prevalence of PD is estimated to be 0.3% in developed countries [1], and the age of onset is typically 50–65 years [1]. Although its main symptom is motor impairment, patients may show symptoms of cognitive impairment and reduced motivation [1].

Interestingly, certain occupations, such as agriculture-related jobs, are known to increase the risk of developing PD, probably due to the effects of pesticides on dopaminergic neurons [2]. In addition, ≥3 independent studies point to an association between medical doctors (and sometimes medical doctors and dentists) and the risk of developing PD [3,4,5], although the underlying causes remain unclear.

Accordingly, patients with PD show several premorbid personality traits and behaviors, including industriousness [6,7], Type A personality [8], low novelty seeking [9], and low habituation to new situations [7]. Moreover, PD premorbid behavior includes low addictive behaviors, such as smoking and alcohol consumption [10,11]. Our previous studies have shown that personality traits, such as novelty seeking and persistence (perseverance despite frustration and fatigue, as well as a tendency toward a persistent pursuit of desired goals), ref. [12] are related to changes in the microstructural properties of dopaminergic areas, such as the putamen and globus pallidus [13], which receive dopaminergic inputs from the substantia nigra [14,15] and play a key role in motivation [16]. These findings indicate that patients with PD show differences in dopamine-related behaviors and personalities even before the onset of the disease.

Previous studies have reported that a higher genetic risk of diseases, such as schizophrenia, is associated with jobs requiring creativity [17]. However, to date, a higher genetic risk of PD has not been associated with any occupation. Thus, this study aimed to determine whether a higher genetic risk of PD is associated with a medical occupation. Our hypothesis is that a higher genetic risk of PD as indicated by a high polygenic risk score (PRS) is associated with career as a medical doctor. This hypothesis is based on the fact that medical doctors are more likely to suffer from PD and that certain premorbid personality traits associated with PD, such as low novelty seeking, are common among medical doctors [18].

## 2. Methods

### 2.1. Participants and Basic Information About the Project

We utilized information obtained from the UK Biobank, a comprehensive dataset originating from a long-term cohort study targeting middle-aged adults in the United Kingdom. Details regarding participant enrollment and data acquisition methods have been documented elsewhere (http://www.ukBiobank.ac.uk/wp-content/uploads/2011/11/UK-Biobank-Protocol.pdf, accessed on 5 July 2021). In summary, individuals between 40 and 69 years of age, listed with the National Health Service and residing within a 25-mile radius of the assessment centers, were invited via email to participate. No specific exclusion criteria were applied during the recruitment process. The current research received ethical clearance from the North–West Multi-centre Research Ethics Committee based in Manchester, UK, and all participants provided written informed consent. Data were collected at one of 22 assessment centers spread across the UK, yielding baseline data from 502,505 individuals. For the analyses presented here, only records with complete information on all required variables, including genetic data, were considered. This section draws significantly on methodology described in our prior publication employing the same approach [19].

### 2.2. Lifetime Job History

Among all 505,205 UK Biobank participants in our project, 120,277 had available information on their lifetime job history, whereas 324,951 had current job information at the first assessment visit. We performed the first analysis using only those with the former information and the second analysis, as a replication, using only those with the latter but without the former information.

A web-based system, Occupations Self-Coding Automatic Recording (OSCAR), was used to examine the employment history of UK Biobank participants. Methodological details have been published previously at great length [20]. In brief, OSCAR is an online categorical decision tree based on a simplified but faithful version of the hierarchical structure of the British Standard Occupational Classification (SOC) v. 2000 [21]. The three-level decision tree used appears as follows: the linked list of jobs on the three webpages starts with 15 main occupational groups (as a proxy for the industrial sector), followed by occupational subgroups and specific job types (i.e., the original 353 four-digit SOC codes), to enable participants to quickly and easily find all jobs they had in their lives. Once the final job title is selected, a hidden four-digit SOC code is automatically assigned to that job title and stored in the database. By design, OSCAR records full-time paid jobs for a minimum of 6 months. The start and end years of each job are recorded and displayed along with the job gap in a timetable that participants can edit at any time, helping to visualize and accurately build a “career timeline”. This description of methods is mostly regenerated from those of a previous study, which is written by the developer of this system [22]. Participants with answers in the categories “medical doctor, general practitioner, and hospital consultant” or “dentist, dental surgeon, orthodontist, and periodontist” were differentiated from those without answers in those categories.

### 2.3. Job Information at the First Assessment Visit

Participants currently employed or self-employed were asked to describe their jobs by the interviewer. The job title is categorized according to SOC v.2000. Detailed information on the interview questions can be found elsewhere (https://biobank.ctsu.ox.ac.uk/crystal/refer.cgi?id=100235, accessed on 1 May 2023). Participants with answers in the categories of “medical practitioners” or “dental practitioners” among health professionals were differentiated from those without answers in those categories.

### 2.4. Education Duration

In addition, we utilized the data on education duration to examine the associations between PD PRS and general education level. Education qualification level data were obtained from the UK Biobank and converted to education duration. This conversion is described in detail in Supplementary Methods.

### 2.5. PD Risk Assessment

We employed the previously described summary statistics of a previous study [23] that focused on the 1805 single-nucleotide polymorphisms (SNPs) that most effectively distinguish PD from control subjects. In brief, Nalls et al. performed a meta-analysis of 17 genome-wide association study datasets on PD, incorporating 37,688 patients, 18,618 proxy cases (i.e., first-degree relatives of PD patients but without PD), and 1.4 million control subjects. The study pinpointed a threshold for calculating the PRS to accurately differentiate patients from controls, involving 1805 genetic variations. Based on the strength of their associations, the SNPs were assigned weights, which were later combined. For this computation, we excluded data that did not pass quality control as well as the data from first-degree relatives of patients with PD included in the above-mentioned previous study [23]. The data from first-degree relatives of patients with PD included in Nalls, Blauwendraat [23] were removed from this study to ensure that any statistical effects overfitted to differences between the two groups did not bias the findings of this study by removing one group. Finally, the score was converted to a Z score (mean is 0 and standard deviation is 1). The full details of the analysis, including the software used, were provided in the supplemental online material. The descriptions in this subsection are largely reproduced from our previous study using the same methods [19]. We also provided the full PRS weights and the list of SNPs used as supplemental online material (Table S1).

### 2.6. Sociodemographic and Lifestyle Measurements Used as Covariates

Age at baseline (cov1, data field ID: 21003), genetically confirmed Caucasoids or non-Caucasoids (cov2, data field ID: 22006), genetic sex (cov3, data field ID: 22001), and 20 genetic principal components (cov 4–23, data field ID: 22009) were extracted from the database. For additional details, refer to Supplementary Methods.

### 2.7. Statistical Analyses

All statistical analyses were performed using Predictive Analysis Software, version 22.0.0 (SPSS Inc., Chicago, IL, USA, 2010).

We used logistic regression analysis to examine the relationship between the PRS for PD and anytime employment as a medical doctor/dentist. In our first analysis, we used data from subjects with a complete job history to examine the relationship between PRS and a career as a medical doctor or dentist; in the second, we used data from subjects without a complete job history to examine the relationship between information on current occupation and PRS for PD. Finally, using data from subjects who had information on their current job with or without job history information, we analyzed the relationship between the PRS for PD and current occupation as a medical doctor or dentist. In these analyses, the 23 variables described above were used as covariates.

We then used partial correlation analyses to examine the relationship between educational history and PD PRS. In these analyses, the variables of cov 1–23, which were described in the sociodemographic and lifestyle measurements used as covariates subsection, were added as covariates. The first and second additional analyses used data from subjects with education duration data from the sample of occupational relationships described above. The third analysis used data from subjects for whom both data on variables 1–23 and education duration were available.

For psychological analyses, results with a threshold of *p* < 0.05 (two-tailed) corrected for false-discovery rate (FDR) using the two-stage sharpened method [24] were considered statistically significant.

## 3. Results

### 3.1. Basic Data

The characteristics of the PD PRS tertiles in each analysis are provided in Table 1.

### 3.2. Association Between PRS for PD and Medical Doctors or Dentists

After correcting for potential confounding factors, we observed that a high PRS for PD significantly and positively correlated with a job history as a medical doctor or dentist [*p* = 0.006, increasing PRS by 1 SD corresponded to increased adjusted odds ratio of 1.064 (1.018–1.113)] (Table 1 and Table 2)].

### 3.3. Association Between Current Job as Medical Doctor or Dentist at the First Assessment Visit and PRS for PD

**Figure 1:**
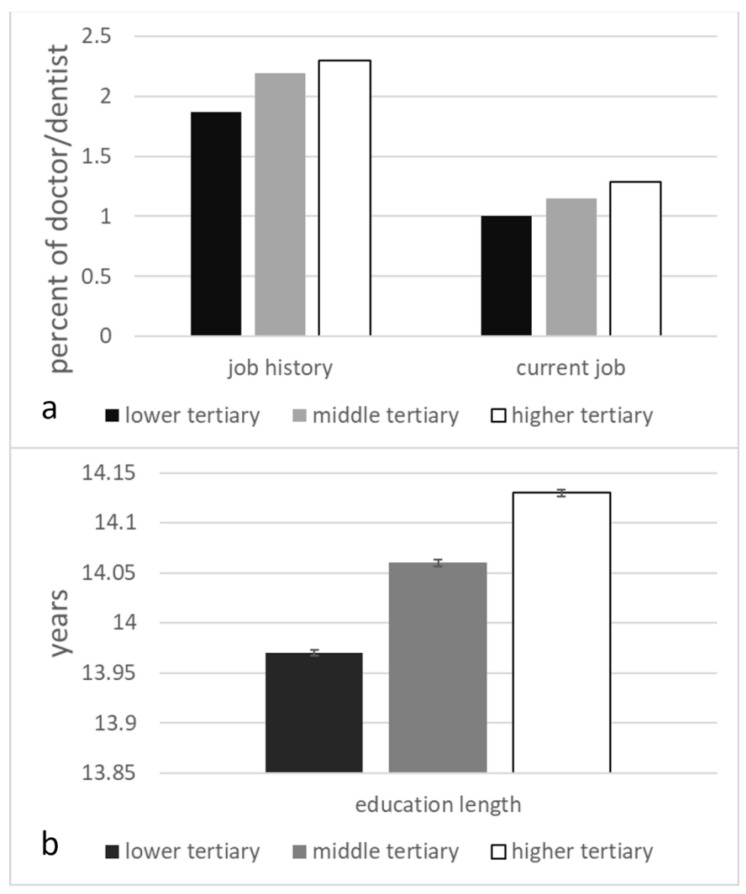
Characteristics of tertiles of PD PRS for each analysis. (**a**) Percentage of doctor/dentist jobs in job history (**b**) as well as percentage of people whose current job is doctor/dentist. Error bars represent the standard error of the mean.

In the first analysis, using data from subjects with job history data, a high PD PRS was associated with having a medical doctor or dentist job history. In the replication analysis, using data from subjects without job history but current job information, a higher PD PRS was associated with a current occupation as a medical doctor or dentist. The third analysis, using data from all subjects with current job information, confirmed a robust association between PD PRS and current status as medical doctor (Table 2, Figure 1a).

### 3.4. Duration of Education

In the first analysis, using data from subjects with job history data, a high PD PRS was associated with longer education. Similarly, in the replication analysis using data from subjects with no job history but current job information, a higher PRS for PD was associated with longer education. In the third analysis using data from all subjects with educational qualifications and covariate data, the PD PRS was robustly related to longer education, as shown in Table 2, as well as in Figure 1b.

## 4. Discussion

This study is the first to show that the overall genetic risk of PD is associated with a medical occupation. This shows that genetic influences can explain at least part of the previously established association between being a medical doctor and a higher risk of PD. The present results indicate that the genetic risk of PD is oriented toward certain professions. Although the evolutionary environment differs in many ways from the modern environment, this may indicate that the PD risk genes might have spread partly because the genetic risk of PD is oriented toward certain important jobs in populations. These associations were obtained from results excluding the first-degree relatives of patients with PD from the analysis, making it less likely that having a close relative with PD would lead to choosing medical doctor as profession.

One possible mechanism underlying this association is that the risk of PD promotes a propensity toward certain personality tendencies through changes in dopamine pathways, which in turn enables employment in occupations that require or have an affinity for hard work. It is known that PD is characterized by premorbid personality traits such as industriousness [6,7], Type A personality [8], and low novelty seeking [9]. Interestingly, higher novelty seeking is associated with substance use in the young and lower academic performance [25], and employment as a medical doctor is known to be associated with lower novelty seeking [18]. PD patients with low novelty seeking tend to have higher persistence in terms of Cloninger’s traits [26]. Persistence means perseverance despite fatigue or frustration [12]. It has been suggested that alterations in dopamine systems are related to both traits [26]. On the other hand, persistence has been reported to show a particularly high positive correlation with academic performance among Cloninger’s traits [27]. Perhaps the genetic risk of PD and low novelty seeking and high persistence may have common dopamine-related physiological bases, and thus, the risk of PRS in PD may be linked to high academic performance and, ultimately, to the profession as a physician. Some of these personality traits, such as low novelty seeking, are related to differences in the microstructural properties of the putamen and globus pallidus [13]. These brain regions receive dopaminergic input from the substantia nigra [14,15] and are said to be involved in motivation [28]. Therefore, the genetic differences associated with the genetic risk of PD in the dopaminergic system may lead to alterations in these systems and lower novelty seeking and high education, as well as the pursuit of a medical career. However, the mechanism of these personality traits is speculative, and the UK Biobank has no information on personality traits related to novelty seeking and diligence. Whether the relationships identified in this study are due to personality traits, as described above, or caused by cognitive differences at younger ages needs to be investigated in future research.

There is at least one limitation of this study. The investigation of this study was conducted using two samples; the samples are limited to residents in the UK. Whether the association observed in this study is universal and independent of cultural, ethnic, and institutional factors needs to be investigated by exploring whether similar associations are present in other regions. Furthermore, we removed participants with missing data from the analysis instead of imputing the missing data, which may change the characteristics of the sample compared with the entire UK Biobank sample. The second limitation of this study is the generalizability of the sample. It has been shown that UK Biobank participants were more likely to live in less socioeconomically deprived areas than nonparticipants [29]. And it has also been shown that compared with the general population, participants were less likely to be obese, to smoke, and to drink alcohol on a daily basis and had fewer self-reported health conditions, and all-cause mortality and cancer incident rates were lower than in the general population of the same age [29]. These results have suggested evidence of a “healthy volunteer” selection bias, and whether the present findings hold true for the general population should be confirmed in future studies. Another limitation of this study is that although the list of covariates includes genetic ancestry and education, it lacks psychosocial and environmental factors that could influence the career choice for medical occupation. These factors might affect the observed associations.

## Figures and Tables

**Table 1:** Characteristics of tertiles of PD PRS in each analysis.

	Lower Tertile of PD PRS	Middle Tertile of PD PRS	Higher Tertile of PD PRS
Participants with job history	31,522	31,521	31,530
Age [mean (SD)]	56.05 (7.69)	55.94 (7.69)	55.93 (7.68)
Education [mean (SD), N]	15.67 (4.72), 31,363	15.75 (4.70), 31,364	15.82 (4.70), 31,366
Medical doctor/dentist	591 (1.87%)	691 (2.19%)	7.25 (2.30%)
Participants with current job info without job history	62,302	62,302	62,320
Age [mean (SD)]	54.06 (7.79)	53.97 (7.84)	53.93 (7.86)
Education [mean (SD), N]	14.05 (5.03), 61,655	14.13 (5.03), 61,655	14.21 (5.05), 61,575
Medical doctor/dentist	415 (0.66%)	460 (0.74%)	546 (0.88%)
All participants with current job info	84,925	84,925	84,950
Age [mean (SD)]	54.08 (7.68)	53.98 (7.71)	53.93 (7.72)
Medical doctor/dentist	855 (1.0%)	976 (1.15%)	1096 (1.29%)
All participants with education variables	128,431	128,428	128,468
Education [mean (SD)]	13.97 (5.13)	14.06 (5.13)	14.13 (5.14)

**Table 2:** Association between PRS for PD and medical doctor/dentist occupation or education duration.

Phenotype	Group	Medical Doctor and Dentist	Not Medical Doctor or Dentist	aOR (95%CI) for Increase of 1 SD	*p* (unc) *	*p* (FDR) **
Medical doctor and dentist	Participants with job history	2007	92,566	1.064 (1.018–1.113)	0.006	0.007
	Participants with current job info without job history	1291	166,531	1.078 (1.021–1.137)	0.006	0.007
	All participants with current job info	2927	251,873	1.077 (1.038–1.118)	9.0 × 10^−5^	2.7 × 10^−4^
Phenotype	Group		Sample size	Beta (95%CI)	*p* (unc)	*p* (FDR)
education	Participants with job history		94,093	0.007 (0.001–0.014)	0.024	0.024
	Participants with current job info without job history		184,785	0.007 (0.003–0.012)	0.001	0.002
	Entire sample with variables		385,327	0.010 (0.007–0.013)	6.8 × 10^−10^	4.1 × 10^−9^

* *p* value of uncorrected. ** *p* value of false discovery rate.

## Data Availability

Researchers can apply to use the UK Biobank resource and access the data used.

## Supplementary Materials

The following supporting information can be downloaded at: https://www.mdpi.com/article/10.3390/genes16040384/s1, Table S1: The list of SNPs, alleles and the effects used for the calculation of PD PRS; Supplemental Methods.
