# Supplementary material for: The Polygenic Risk Score for Parkinson’s Disease Is Associated with Becoming a Medical Doctor or Dentist
Source: Genes (Basel). 2025 Mar 28;16(4):384. doi: 10.3390/genes16040384 (PMC12026780; doi:10.3390/genes16040384)
Supplement: Supplementary file 1 [file genes-16-00384-s001.zip › ukb_parkinPRSjobs_SOM_gene_submitted.pdf]

Supplementary online material

Supplemental Methods

Additional information on subjects

Adults aged 40–69 years who were registered with the National Health Service (NHS) and resided within 25 miles of the study evaluation sites were invited through email to participate in the UK Biobank project. No exclusion criteria were applied for this recruitment. The descriptions here are reproduced from our previous study using the exact same methods (Takeuchi and Kawashima, 2022b).

Education length

Education level was based on self-reported data. Education level categories were transformed into the following numerical values (in years) as previously described (3): "College or University degree" = 20 years; "A levels/AS levels or equivalent" = 13 years; "O levels/GCSEs or equivalent" = 10 years; "CSEs or equivalent" = 10 years; "NVQ or HND or HNC or equivalent" = 19 years; "Other professional qualifications e.g.,: nursing, teaching, etc." = 15 years; "None of the above" = 7 years; and "Prefer not to answer" = missing. This value was obtained at recruitment and used for all analyses. The description

in this study was reproduced from our previous study using the exact same methods (Takeuchi and Kawashima, 2022a).

Genetic principal components

We used the genetic principal components provided by the UK Biobank (data-field IDs: 22009), calculated using unrelated, high-quality samples and 147,604 high-confidence markers after pruning for linkage disequilibrium. Methodological details are available elsewhere (https://biobank.ctsu.ox.ac.uk/crystal/refer.cgi?id=590).

Details of polygenic risk score (PRS) calculation

We followed the procedures described by Nalls*, et al.* (Nalls et al., 2019), a published pipeline from the UK Biobank (Collister et al., 2022), and the homepage accompanying a previous study (https://2cjenn.github.io/PRS_Pipeline/) for calculating the PRS of PD. The descriptions here are mostly based on these sources. Genome-wide association study (GWAS) statistics of Parkinson's disease were provided by the first author of (Nalls et al., 2019). We excluded UK Biobank participants with data missing from a specific field (Field 22020) as this indicated that the data did not meet internal quality control checks for inclusion in the calculation of principal components. This

quality control includes (a) a missing rate in autosomes of ≤0.02, (b) not outliers for missingness or heterozygosity, (c) not in a maximal set of unrelated individuals, and (d) not sex-discordant.

In total, 1805 independent allelic variants were used to calculate the PRS without linkage disequilibrium (LD) clumping or P thresholding. For each participant of the UK Biobank, the number of associated alleles weighted by the beta of the summary statistics of Nalls*, et al.* (Nalls et al., 2019) was counted and summed across all SNPs using the --score command in PLINK2. The no-mean-imputation option was also used to scale each individual's score according to the number of non-missing SNPs without imputing missing SNPs.

In the GWAS used in Nalls et al. (Nalls et al., 2019), the first-degree relatives of patients with PD were used as proxy cases to estimate the associations between SNPs and PD. Thus, we removed these subjects from the PRS analysis.

References

Collister, J.A., Liu, X. and Clifton, L., 2022. Calculating Polygenic Risk Scores (PRS) in UK Biobank: A Practical Guide for Epidemiologists. Frontiers in Genetics 13, 818574-818574.

Nalls, M.A., Blauwendraat, C., Vallerga, C.L., Heilbron, K., Bandres-Ciga, S., Chang, D., Tan, M., Kia, D.A., Noyce, A.J. and Xue, A., 2019. Identification of novel risk loci, causal insights, and heritable risk for Parkinson's disease: a meta-analysis of genome-wide association studies. The Lancet Neurology 18, 1091-1102.

Takeuchi, H. and Kawashima, R., 2022a. Effects of Body Mass Index on Brain Structures in the Elderly: Longitudinal Analyses. Frontiers in endocrinology 13, 824661.

Takeuchi, H. and Kawashima, R., 2022b. Effects of Diastolic Blood Pressure on Brain Structures and Cognitive Functions in Middle and Old Ages: Longitudinal Analyses. Nutrients 14, 2464.
